# Validity of Online Screening for Autism: Crowdsourcing Study Comparing Paid and Unpaid Diagnostic Tasks

**DOI:** 10.2196/13668

**Published:** 2019-05-23

**Authors:** Peter Washington, Haik Kalantarian, Qandeel Tariq, Jessey Schwartz, Kaitlyn Dunlap, Brianna Chrisman, Maya Varma, Michael Ning, Aaron Kline, Nathaniel Stockham, Kelley Paskov, Catalin Voss, Nick Haber, Dennis Paul Wall

**Affiliations:** 1 Department of Bioengineering Stanford University Stanford, CA United States; 2 Department of Biomedical Data Science Stanford University Stanford, CA United States; 3 Department of Computer Science Stanford University Stanford, CA United States; 4 Department of Neuroscience Stanford University Stanford, CA United States; 5 Department of Pediatrics Stanford University Stanford, CA United States; 6 Department of Psychology Stanford University Stanford, CA United States; 7 Department of Psychiatry and Behavioral Sciences Stanford University Stanford, CA United States; 8 Division of Systems Medicine Department of Biomedical Data Science Stanford University Palo Alto, CA United States

**Keywords:** crowdsourcing, autism, mechanical turk, pediatrics, diagnostics, diagnosis, neuropsychiatric conditions, human-computer interaction, citizen healthcare, biomedical data science, mobile health, digital health

## Abstract

**Background:**

Obtaining a diagnosis of neuropsychiatric disorders such as autism requires long waiting times that can exceed a year and can be prohibitively expensive. Crowdsourcing approaches may provide a scalable alternative that can accelerate general access to care and permit underserved populations to obtain an accurate diagnosis.

**Objective:**

We aimed to perform a series of studies to explore whether paid crowd workers on Amazon Mechanical Turk (AMT) and citizen crowd workers on a public website shared on social media can provide accurate online detection of autism, conducted via crowdsourced ratings of short home video clips.

**Methods:**

Three online studies were performed: (1) a paid crowdsourcing task on AMT (N=54) where crowd workers were asked to classify 10 short video clips of children as “Autism” or “Not autism,” (2) a more complex paid crowdsourcing task (N=27) with only those raters who correctly rated ≥8 of the 10 videos during the first study, and (3) a public unpaid study (N=115) identical to the first study.

**Results:**

For Study 1, the mean score of the participants who completed all questions was 7.50/10 (SD 1.46). When only analyzing the workers who scored ≥8/10 (n=27/54), there was a weak negative correlation between the time spent rating the videos and the sensitivity (ρ=–0.44, *P*=.02). For Study 2, the mean score of the participants rating new videos was 6.76/10 (SD 0.59). The average deviation between the crowdsourced answers and gold standard ratings provided by two expert clinical research coordinators was 0.56, with an SD of 0.51 (maximum possible SD is 3). All paid crowd workers who scored 8/10 in Study 1 either expressed enjoyment in performing the task in Study 2 or provided no negative comments. For Study 3, the mean score of the participants who completed all questions was 6.67/10 (SD 1.61). There were weak correlations between age and score (*r*=0.22, *P*=.014), age and sensitivity (*r*=–0.19, *P*=.04), number of family members with autism and sensitivity (*r*=–0.195, *P*=.04), and number of family members with autism and precision (*r*=–0.203, *P*=.03). A two-tailed *t* test between the scores of the paid workers in Study 1 and the unpaid workers in Study 3 showed a significant difference (*P<*.001).

**Conclusions:**

Many paid crowd workers on AMT enjoyed answering screening questions from videos, suggesting higher intrinsic motivation to make quality assessments. Paid crowdsourcing provides promising screening assessments of pediatric autism with an average deviation <20% from professional gold standard raters, which is potentially a clinically informative estimate for parents. Parents of children with autism likely overfit their intuition to their own affected child. This work provides preliminary demographic data on raters who may have higher ability to recognize and measure features of autism across its wide range of phenotypic manifestations.

## Introduction

Autism spectrum disorder (ASD, or autism) [1] is a complex neurodevelopmental disorder that manifests in children by or before 3 years of age and now impacts 1 in 40 children in the United States [2]. Obtaining a diagnosis of ASD, like that of many other neuropsychiatric conditions, requires long waiting times, often exceeding a year [3,4]. Furthermore, obtaining a professional diagnosis is often prohibitively expensive for much of the global population [5,6]. *Crowdsourcing* provides a scalable alternative to the current diagnostic evaluations for ASD that include extensive and lengthy clinical evaluation by a trained professional and are inaccessible to families in rural areas as well as those with low incomes [7]. Crowdsourcing, broadly defined, is a type of participative online activity in which an entity proposes the voluntary undertaking of a task to a group of individuals [8]. Crowdsourcing can improve the quality and speed of medical research projects by leveraging the crowd for large-scale problem solving, data processing, surveillance/monitoring, and surveying [9]. Thus, crowdsourcing may enable families who are limited by long waiting times, are members of underserved populations in the United States, or are living in countries where accessibility is hampered by available resources to obtain an accurate diagnosis in a timely manner.

Crowdsourcing is increasingly used in health promotion, research, and care [10]. Ben-Sasson et al showed that parents of children suspected to have autism can fill out standardized questionnaires about their own child online to prescreen for autism; a machine learning classifier trained on the questionnaire responses identified 58% to 88% of children at risk for autism [11]. Tariq et al showed that feature tagging by independent nonexpert raters enables rapid machine learning risk prediction for autism by using home videos of <3 minutes in length [12]. However, such approaches have not yet been performed or tested at scale.

Large-scale crowdsourcing can be achieved through online and virtual workforce platforms. Amazon Mechanical Turk (AMT) is a popular crowdsourcing platform that offers a paradigm for engaging a large number of users for short times and low monetary costs [13]. AMT has been successfully used in health care settings. Kuang et al showed that crowd workers on AMT had significantly higher scores when rating pictographs than in-person participants in a hospital [14]. CrowdMed is a software platform that aims to leverage crowdsourcing to help undiagnosed or misdiagnosed patients by allowing them to submit their cases and interact with case solvers to obtain diagnostic outcomes [15]. Such large-scale crowdsourcing systems and paradigms have not yet been tested for their potential in assisting the screening and diagnostic processes for children at risk for developmental delays.

A limiting factor for the crowdsourcing detection of pediatric conditions is the collection of structured data such as video or audio. Recently, an increasing number of mobile health tools are being developed for children with autism [4,16-25], including at least one that has been clinically validated in a randomized controlled trial [26]. These tools not only provide opportunities for better health care but can also be a significant data resource and specifically increase the potential to collect rich, naturalistic behavioral data via structured mobile videos [27,28]. Nazneen et al developed an effective system used in a home setting to capture videos of children with autism, and professional diagnosticians deemed 96% of the collected videos clinically useful for making an autism diagnosis [29]. Voss et al developed a wearable system on Google Glass providing real-time emotion feedback to the child wearer while simultaneously capturing videos through the front-facing camera of the Glass [19,24-26]. Kalantarian et al developed a mobile charades game that promotes facial contact with the parent by the affected child while capturing highly structured videos through the front-facing camera of iOS and Android devices [27,28]. Videos produced by such systems can be fed into a crowdsourcing pipeline for manual labeling of videos that can be analyzed using artificial intelligence and produce rapid screening and diagnosis for children at risk for developmental delay and other conditions marked by behavioral symptoms. Such applications could be particularly valuable because they have the potential to flow more easily into the health care system, given the widespread adoption of mobile devices globally [30,31], enabling easy and free data collection and transfer between families, crowdsourced workforces, and the health care system.

Here, we present a series of three crowdsourced studies which enabled us to (1) test the ability of the crowd to directly identify autism and (2) provide behavioral metrics that could be used for machine learning autism classification based on a short video clip of a child interacting with his/her parent. In Study 1, we evaluated whether a randomly selected set of paid crowd workers could accurately label videos of children interacting with family members as either “Autism” or “Not autism.” In Study 2, we evaluated whether high-scoring crowd workers providing intuitive answers about a disorder would perform well on a different set of videos and be motivated to perform a more thorough task on AMT. In Study 3, we tested how unpaid crowd workers perform when rating videos for diagnostics. We hypothesized that the workers would enjoy the rating task, certain demographics of workers would emerge as high-quality raters, paid and unpaid crowd workers would perform equally well on the same set of videos, high-scoring workers on simple rating tasks would continue to perform well on harder rating tasks, and crowd ratings would approximate a set of “gold standard” ratings from professionals.

## Methods

### Summary of Studies

We performed three studies that were designed incrementally in response to the results from our prior work, which examined feature tagging by independent nonexpert raters for autism risk prediction using home videos [12]. All three studies were approved by the Stanford Institutional Review Board. Videos for all studies were sourced from publicly available YouTube videos. We searched for videos of children both with and without autism based on the following criteria for the videos: (1) it shows the child’s hands and face; (2) it includes clear opportunities for direct social engagement; and (3) it involves opportunities for the child to use an object such as a utensil, crayon, or toy. We selected a subset of 20 videos for this study balanced by age, gender, and diagnosis. Table 1 provides video demographic statistics of the sets of videos used in all tasks.

### Study 1: Paid Crowdsourcing on Mechanical Turk

#### Overview

In order to evaluate whether a randomly selected set of paid crowd workers could accurately classify videos as either “Autism” or “Not Autism,” we recruited 54 workers on AMT and recorded their demographic traits.

#### Participants

Workers were paid US $3.50 each to complete the task, which aligns with the California minimum wage payment rate based on our estimate of the time needed to complete each task. To ensure quality, workers were required to have a task approval rate >98% for all requesters’ human intelligence tasks and a total number of approved human intelligence tasks >500. In order to identify any differences in rating ability based on demographic trends, we asked workers for their age and gender, whether the rater is a parent, the number of children the rater knows with autism, the number of family members with autism, the number of affected friends, and whether the rater himself/herself has autism.

#### Task

**Figure 1:**
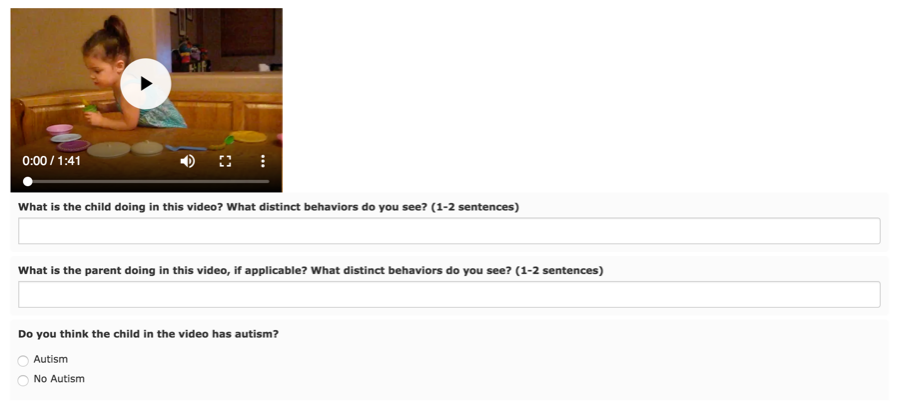
An example question set on the paid crowdsourcing Mechanical Turk Study 1 task. Workers answered the same set of questions for 10 separate videos.

The task consisted of workers viewing and answering questions on 10 videos of a parent interacting with a child. The videos were equally balanced for gender and diagnosis (Table 1). Workers were not required to watch the entire video but were instructed to *“scroll through the video once you get the idea, but watch enough to be able to answer the questions*.” We allowed the workers to skip to other parts of the videos and replay them. As shown in Figure 1, each video was followed by free-response questions asking the rater to briefly describe the activity of the parent and the child in the video. This was used to verify that the workers adequately engaged with the videos before classifying them as “Autism” or “No Autism.” If the answers were logically inconsistent, we discarded the worker’s answers. After answering all questions, we asked the workers a series of demographic questions to help them describe themselves. These questions included age, gender, geographic location, whether the rater is a parent, the number of children the rater knows with autism, the number of family members with autism, the number of affected friends, and whether the rater himself/herself has autism (Multimedia Appendix 1).

**Table 1:** Summary of the videos used in all three studies.

Studies	Video length, mean (range)	Child age (years), mean (range)	Female, %	Children with autism, %
1, 2, 3	3 minutes 2 seconds (49 s to 6 min 39 s)	3.2 (2-5)	50	50
2	2 minutes 9 seconds (1 min 7 s to 4 min 40 s)	2.9 (2-5)	50	50

#### Analysis

We used the Pearson correlation when comparing real numbers to performance metrics and the point biserial correlation when comparing binary variables to performance metrics. In particular, Pearson correlation was used to compare scores, precision, recall, and specificity to time spent, age, number of children known with autism, number of family members with autism, number of friends with autism, number of people known with autism, and number of children known with autism. Point biserial correlation was used to compare scores, precision, recall, specificity, and time spent to whether the rater has autism, whether the rater is a parent, and the gender of the rater. The metrics used were accuracy, precision (true positive/[true positive+false positive]), recall (true positive/[true positive+false negative]), and specificity (true negative/[true negative+false positive]). We analyzed the subset of workers who scored well (≥8/10) in addition to the pool of all workers in order to determine demographic traits specific to high-performing workers.

### Study 2: Paid Crowdsourcing With High-Scoring Workers

#### Overview

In order to evaluate whether high-scoring workers providing intuitive answers about a disorder would perform well on a different set of videos and be motivated to perform a more thorough task on AMT, we conducted a follow-up study with the workers who performed well (scored ≥8/10) in Study 1. The study was divided into two parts: (1) conducting the same task as that in Study 1 but with a different set of 10 videos, and (2) answering a series of 31 multiple-choice questions about specific behaviors of the child for each of the 10 videos from Study 1.

#### Participants

A total of 27 workers who scored ≥8/10 in Study 1 were successfully recruited to complete an additional set of 11 tasks. We chose to exclude workers who did not perform well in Study 1 because we wanted to filter out workers who did not demonstrate intuitive skill for detecting developmental delays in children. We chose a cutoff of 8/10 because a higher cutoff would not yield a large enough worker pool to recruit from. Workers were recruited by providing a worker bonus of US $0.05, and they were sent a message describing the additional tasks and pay for completion of the tasks.

#### Tasks

**Figure 2:**
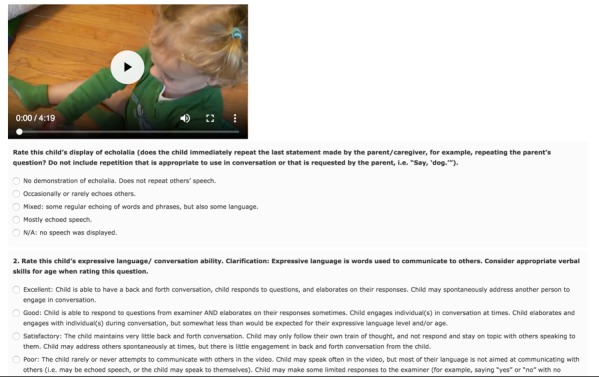
Two questions on the paid crowdsourcing Amazon Mechanical Turk Study 2 multiple-choice tasks. Workers were asked to answer 31 multiple-choice questions for a single video per task. There were 10 available identical tasks with different videos.

In the first task, the setup was identical to that in Study 1 except that a different set of videos was used (Table 1) *.* The remaining 10 tasks required workers to answer a series of 31 multiple-choice questions that have been previously shown to have high predictive power for detecting autism through video [12]. The 31 multiple-choice questions target 31 different symptoms of ASD and are written to rate the presence of these symptoms in short videos of children. Each question asks the rater to rate an individual symptom of autism, with the answer choices representing increasing levels of severity; each question therefore serves as a rating scale that allows for quantitative comparisons. The full set of 31 questions can be found in a previous study [12]. To understand the worker satisfaction with the tasks, we asked all workers to provide any free-form comments about the rating experience. Figure 2 shows the user interface for these tasks. The same set of 10 videos used in Study 1 was also used in this series of tasks. For each task, the interface consisted of a single video followed by a series of 31 multiple-choice questions, including the original diagnosis question (“Autism” or “Not autism”) asked in Study 1. Because all the recruited workers had already performed well in Study 1, no verification questions about the video were asked.

#### Comparison to Gold Standard Ratings

In order to compare the answers provided by the crowd workers with a “gold standard” rating, we asked two trained clinical research coordinators experienced in working with children with autism and neuropsychiatric disorders to answer all 31 questions for each of the 10 videos that included multiple-choice questions. This rating was used as a baseline to compare the answers from the AMT workers.

#### Analysis

As in Study 1, Pearson correlation was used to compare scores to time spent, age, number of children known with autism, number of family members with autism, number of friends known with autism, number of people known with autism, and number of children known with autism. Point biserial correlation was used to compare scores to whether the rater has autism, whether the rater is a parent, and the gender of the rater.

### Study 3: Public Crowdsourcing Through Citizen Healthcare

#### Overview

In order to test how unpaid crowd workers perform when rating videos for diagnostics, we developed a public website (videoproject.stanford.edu) for watching the videos and answering questions about the videos. Through pilot testing, we found that unpaid crowd workers are not willing to answer 31 multiple-choice questions for several videos; therefore, we focused on the "Autism or Not" task from Study 1.

#### Participants

A total of 115 participants were successfully recruited via our public-facing website (videoproject.stanford.edu) that was distributed via social media shares and online community noticeboards (eg, Nextdoor.com).

#### Task

**Figure 3:**
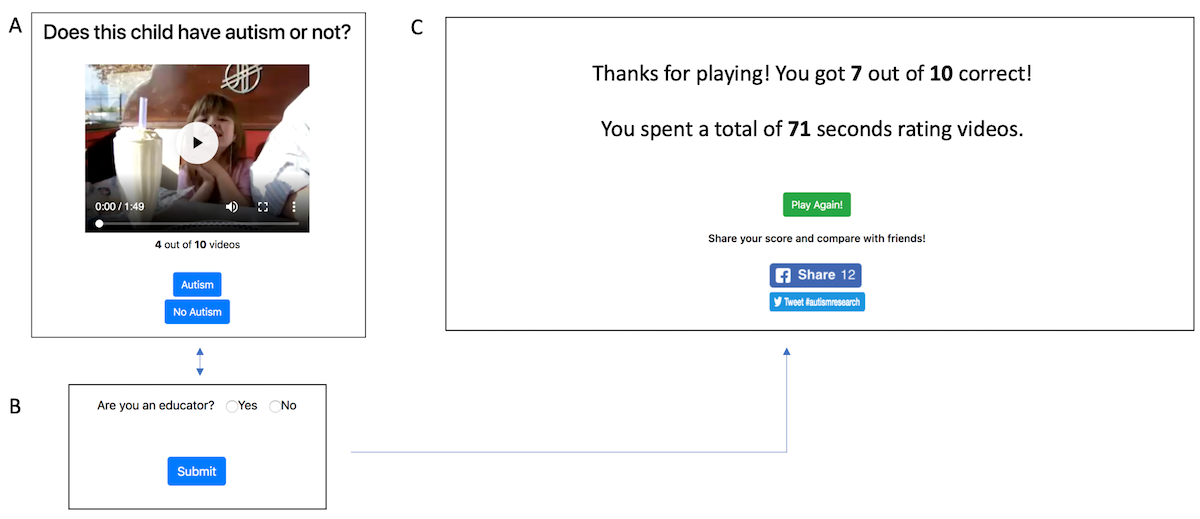
(A) The primary interface for the "citizen healthcare" public crowdsourcing study. Citizen healthcare providers watch a short video and then classify the video as "Autism" or "Not Autism." (B) After rating each video in the "citizen healthcare" public crowdsourcing study, users are asked a single demographic question about themselves. This allows us to collect demographic information without overwhelming the user, which would otherwise lead to lower participant retention rates. (C) At the end of the "citizen healthcare" public crowdsourcing study, users are informed of their score and the time they spent rating. They then have the option to play the game again and share their result on Facebook or Twitter.

When the users navigated to the webpage, they were provided with a video and two buttons allowing them to classify the video as “Autism” or “Not autism” (Figure 3A), as in Study 1. In order to minimize participant dropout due to a long list of demographic questions, we interleaved each question throughout the video-rating process (Figure 3B). After rating all videos and providing all demographic information, the user is directed to a results page where his/her score and total rating time are displayed along with the option to play again and share their results on social media (Figure 3C).

## Results

### Study 1: Paid Crowdsourcing on Mechanical Turk

The mean score of the participants who completed all questions was 7.5/10 (SD 1.46). Table 2 shows the summary demographics of the crowd workers who completed the task. Most of the demographic trends from the Mechanical Turk cohort were not statistically significant. However, when only analyzing the workers who scored well (scored ≥8/10; N=27/54), there was a weak negative correlation between the time spent rating the videos and the sensitivity (ρ=–0.44, *P*=.02). There were similar almost-significant trends for accuracy (ρ=–0.35, *P*=.07) and precision (ρ=–0.38, *P*=.05). There was no trend for sensitivity or specificity. Table 3 shows the average rating of all video raters.

**Table 2:** Summary demographics of the crowd workers in Study 1 (N=54).

Demographic	Value
Age, mean (SD)	36.4 (9.0)
With autism, n (%)	3 (5.6)
Is a parent, n (%)	25 (46.3)
Female, n (%)	20 (37.0)
Number of known affected children, mean (SD)	0.7 (0.9)
Number of affected families, mean (SD)	0.4 (0.7)
Number of affected friends, mean (SD)	1.3 (1.2)
Number of total known affected people, mean (SD)	2.3 (3.3)

**Table 3:** Ratings labeled as “Autism” across all 54 paid crowd workers in Study 1.

Video number	Ratings labeled as “Autism”, %	True rating
1	87	Autism
2	6	Not autism
3	2	Not autism
4	44	Autism
5	81	Autism
6	2	Not autism
7	39	Autism
8	49	Not autism
9	70	Autism
10	2	Not autism

### Study 2: Paid Crowdsourcing With High-Scoring Workers

#### Overview

Table 4 shows the comparison between workers who performed well (≥8/10 videos correctly diagnosed) and poorly (<8/10) in Study 1. There were no statistically significant differences between the two populations except in the mean number of affected children that the worker knew.

#### Performance on Different Video Sets

The mean score of the crowd workers was 6.76/10 (SD 0.59). Because the study cohort was smaller for Study 2, we did not analyze demographic trends. Instead, we analyzed completion rate, rating trends, and agreement with “gold standard” raters. Table 5 shows the autism classification ratings of all raters in this part of the study. There was significantly more rater agreement than that in Study 1, indicating that crowd workers who perform well on providing diagnoses on one set of videos will also perform well on a different set of videos with similar characteristics.

#### Worker Satisfaction

None of the workers provided any negative comment about any of the tasks in this study. Several workers had positive comments (Textbox 1).

#### Worker Motivation

In addition to thanking the researchers for the provided tasks, some workers (4/27) volunteered detailed explanations about the videos and the reasoning behind their ratings. Comments from Video 4 are shown as a representative example in Textbox 2.

**Table 4:** Comparison of summary demographics of the crowd workers who performed well (≥8/10 videos correctly diagnosed) and poorly (<8/10) in Study 1 (N=27).

Demographic	Performed well (score≥8/10)	Performed poorly (score<8/10)	*P* value
Age, mean (SD)	34.7 (6.5)	38.1 (10.8)	.17
With autism, n (%)	2 (7.4)	1 (3.7)	.56
Is a parent, n (%)	12 (44.4)	13 (48.1)	.79
Female, n (%)	12 (44.4)	8 (29.6)	.27
Number of known affected children, mean (SD)	0.5 (0.7)	1.0 (1.0)	.048
Number of affected families, mean (SD)	0.2 (0.4)	0.5 (0.9)	.09
Number of affected friends, mean (SD)	1.1 (1.3)	1.5 (1.2)	.23
Number of total known affected people, mean (SD)	2.3 (3.9)	2.3 (2.6)	0.97

**Table 5:** Ratings labeled as “Autism” across all 22 paid crowd workers in the task with a different set of 10 videos.

Video number	Ratings labeled as “Autism”, %	True rating
11	100	Autism
12	0	Not autism
13	43	Autism
14	0	Not autism
15	90	Autism
16	76	Autism
17	90	Autism
18	10	Not autism
19	24	Not autism
20	0	Not autism

**Representative examples of positive comments from crowd workers.**

*"Well organized and enjoyable.”*

*"Thank you as always! I appreciate the opportunity and the behavioral learning experience.”*

*"Fantastic survey. I really hope there are more of these in the future!”*

*"Thank you so much! So glad to be a part of your studies and hope that this will progress your work in autism.”*

*"Thank you and look forward to more hits.”*

**Explanations for the ratings for Video 4.**

*"It was a bit hard to hear the other people besides the child, so not sure if they were talking to her when she shouted 'yes.'”*

*"I want to say this child has Autism, but would like to see some more sensory information before I truly decided. Hence why some questions were N/A.”*

*"The child seems to be listening to something on earphones. Some part of it seems like she is singing along to it. The limited interaction with parents seems more like distraction by the music than some developmental problem.”*

*"While I think this girl has some sort of developmental issues, (her playing with the straw, hand motions, fixation on the parents phone at the end), I think it's a stretch to call it autism. I'll do the last one of these later today!”*

#### Comparison with Gold Standard Ratings

**Figure 4:**
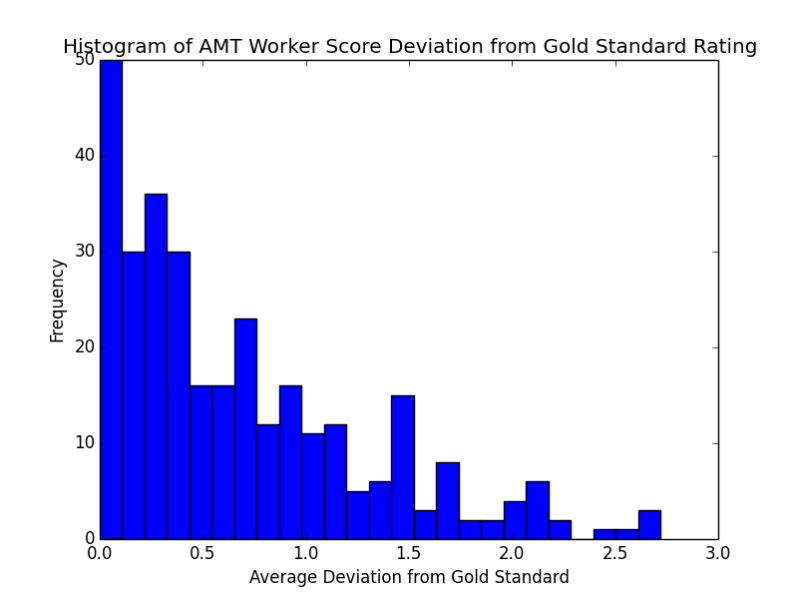
A histogram of the AMT worker deviation from the gold standard ratings for all questions and all videos. The maximum possible deviation is 3.0. Most video ratings have a deviation below 1.0, which is an acceptable error. However, several worker responses deviated greatly from the gold standard. AMT: Amazon Mechanical Turk.

The ratings between the two gold standard raters were identical for all videos except for one, where the answers differed by one point for a single question. Across all videos, the average deviation between the average crowdsourced answers and the gold standard ratings was 0.56, with an SD of 0.51. Figure 4 shows the distribution of deviations of all questions for all videos. There were 310 data points (31 questions × 10 workers). We followed up this analysis with a qualitative inspection of all video-question pairs where the average deviation exceeded 1.5.

#### Analyzing the Underlying Cause of Worker Deviation

A qualitative analysis of the video-question pairs where the average deviation exceeded 1.5 answer choices on the rating scale helped us explore the underlying cause of worker deviation from the gold standard rating. There were 22 such pairs (of a possible 31×10=310 pairs). There were 2 questions, in particular, that had high deviation across multiple videos (Table 6). Questions 13 and 16 were vaguer than the other questions, providing a list of numerous example behaviors within the question. These example behaviors were not exhaustive; therefore, it is possible that some workers only looked for the explicitly listed behaviors without generalizing them. On one video in the dataset, in particular, raters performed poorly on several questions. This video involved a 4-year-old girl with ASD singing songs with her father. It is possible that ASD features are more difficult to distinguish from a video when observing singing rather than natural speech.

**Table 6:** Questions where the average worker answer was >1.5/3.0 answer choices away from the gold standard rating for multiple videos.

Question		Number of deviating videos (of 10)
13	Does the child get upset, angry or irritated by particular sounds, tastes, smells, sights or textures?	4
16	Does the child stare at objects for long periods of time or focus on particular sounds, smells or textures, or like to sniff things?	5

### Study 3: Public Crowdsourcing Through Citizen Healthcare

There were 145 unique visits to videoproject.stanford.edu. A total of 126 participants provided at least one rating of the series of 10 videos. Of these 126 participants who started the rating process, 115 completed all videos (91.3% retention). The mean score of the participants who completed all questions was 6.67/10 (SD 1.61). The mean score of the paid participants was 7.50 (SD 1.46) for the same set of videos in Study 1 and 6.76 (SD 0.59) in Study 2 for a different set of videos with the high-scoring workers. A two-tailed *t* test between the scores of the paid workers in Study 1 and the unpaid workers in Study 3 showed significant differences (*t*_*168*_=3.37, *P*<.001).

As in Study 1, we analyzed the Pearson correlation when comparing real numbers to scores and the point biserial correlation when comparing binary variables to scores. There were weak correlations between age and score (*r*=0.22, *P*=.02), age and sensitivity (*r*=–0.19, *P*=.04), age and total time spent rating videos (*r*=0.25, *P*=.004), whether the rater is an educator and total time spent rating videos (*r*=–0.185, *P*=.045), number of family members with autism and sensitivity (*r*=–0.195, *P*=.04), and number of family members with autism and precision (*r*=–0.203, *P*=.03).

## Discussion

### Interpretation of Principal Results

We have demonstrated the feasibility of both paid and volunteer “citizen healthcare” crowd workers to provide pediatric diagnostic information on behavioral disorders based on short video clips. We first ran a study (Study 1) with 54 AMT workers and found that there was a weak negative correlation between the time spent rating the videos and the sensitivity (ρ=–0.44, *P*=.02). We then conducted a follow-up study (Study 2) with the AMT workers who performed well (correctly classified at least 8 of the 10 videos) in Study 1 and received exclusively positive feedback about the tasks; we even received requests for more. This feedback, in conjunction with the high completion rate for the set of tasks, indicates that crowd workers who perform well in simple tasks (eg, from Study 1) are likely to not only participate in but also *enjoy* completing further tasks if they are encouraged about their prior performance and paid sufficiently.

We also found that across all videos, the average deviation between the average crowdsourced answers and the gold standard ratings was 0.56, with an SD of 0.51. Since the scales are from 0 (not severe) to 3 (severe), this deviation indicates that the crowd tends to rate within acceptable error. Most of the deviations fell within 1.0, although there was a nonnegligible number of video questions with a larger SD.

Finally, we ran the procedures from Study 1 on a public website advertised on social media and found weak correlations between certain demographic groups, due, at least in part, to the small sample sizes per category. Larger sample sizes will be required to draw significant conclusions about the inherent accuracy within or across demographic groups. A two-tailed *t* test between the scores of the paid workers in Study 1 and the unpaid workers in Study 3 showed a significant difference (*t*_*168*_=3.37, *P*<.001). This indicates that paid workers will outperform a general unpaid crowd of online citizen workers.

### Limitations

A limitation of this work includes the lack of the assessment of this crowdsourced “citizen healthcare model” in a real-world clinical setting. We are working on establishing the infrastructure to test this kind of system prospectively (see Future Work). Our current findings using publicly available YouTube videos and “uploader reported” diagnoses for this initial study lend support to the potential for such future research.

It is unclear whether results from AMT can be generalized to all paid crowdsourcing platforms. It is possible that another paid crowdsourcing platform could yield workers with higher or lower performance than those that chose to participate in our AMT studies. There were 27 well-performing workers who moved on to participate in Study 2, but testing Study 2 procedures with participants who scored <8/10 would provide additional insights into the performance of crowdsourced video raters.

We emphasize that the work performed here is a pilot study for crowdsourcing acquisition of pediatric diagnostic information from an untrained population. In future studies, it will be fruitful to explore a larger diagnostic workforce and replicate the processes described here with independent subsets of the crowd.

In terms of the volunteer-based “citizen healthcare” experiment included in this study, some of the results could have been skewed by our recruiting methodologies. We recruited participants largely via conference presentations and recruitment postings on Nextdoor [32] in the San Francisco Bay Area, California, as well as south Austin, Texas. We were ultimately only able to enroll 71 people to complete the study, but a larger-scale crowdsourced study with broader public recruitment strategies might yield emerging demographic trends that did not arise in this study due to the limited sample size.

### Future Work

Future work should examine the potential of crowd workers to provide ratings about other demographic groups such as adults, individuals with other neuropsychiatric disorders, and populations in other geographic regions. Although performing a study on a public cohort of citizen raters scoring 31 multiple questions was not feasible at scale, we believe that future work should explore motivations, through mediums such as gamification, for crowd workers to participate in diagnostic microtasks for free.

Additionally, we hope to assess the feasibility of this pipeline for standard of care practice, where we use the crowd to analyze videos of children referred to developmental specialists by primary care providers. This will not only allow us to better understand the feasibility of using this system in a clinical setting but will also allow us to better assess the validity of the pipeline by utilizing videos of children who receive professional diagnoses. This will permit us to compare diagnostic outcomes from the crowd to those assigned by licensed professionals. In addition, efforts should be made into expanding the source of gold standard ratings to a larger network of expert clinical raters.

Crowdsourcing of rich video data opens the doors to understanding the forms of autism, including the potential contributions from genetics and environment, in part, due to the ability to develop an online community network and a rich digital phenotype for many subjects in a scalable and affordable fashion. Eventually, crowdsourcing could provide scientists with enough data to find the link between genetics and the behaviors present in videos [33]. This work is a step toward this goal.

Using a crowd of raters to answer questions about short structured videos of a child for mobile machine learning-aided detection and diagnosis may help to ameliorate some of the inefficiencies with the current standards of care for autism diagnosis. For families lacking the financial resources to obtain a formal diagnosis, a crowdsourced paradigm like the one tested here could be a viable alternative when provided with a proper system and feature measurement design.

### Conclusion

In summary, we have shown that paid crowd workers enjoy answering screening questions from videos, suggesting higher intrinsic motivation for making quality assessments. Paid and vetted crowd workers also showed reasonable accuracy with detection of autism as well as other developmental delays in children between 2 and 5 years of age, with an average deviation <20% from professional gold standard raters, whereas parents of children with autism likely overfit their video assessments to their own affected child. These results show promise for the potential use of virtual workers in developmental screening and provide motivation for future research in paid and unpaid crowdsourcing for the diagnosis of autism and other neuropsychiatric conditions.

## Appendix

Multimedia Appendix 1 The full list of multiple-choice questions asked on Amazon Mechanical Turk.

## Abbreviations

AMT: Amazon Mechanical Turk
ASD: autism spectrum disorder
