# Addendum to the Acknowledgements: Validity of Online Screening for Autism: Crowdsourcing Study Comparing Paid and Unpaid Diagnostic Tasks

**DOI:** 10.2196/14950

**Published:** 2019-06-27

**Authors:** Peter Washington, Haik Kalantarian, Qandeel Tariq, Jessey Schwartz, Kaitlyn Dunlap, Brianna Chrisman, Maya Varma, Michael Ning, Aaron Kline, Nathaniel Stockham, Kelley Paskov, Catalin Voss, Nick Haber, Dennis Paul Wall

**Affiliations:** 1 Department of Bioengineering Stanford University Stanford, CA United States; 2 Department of Biomedical Data Science Stanford University Stanford, CA United States; 3 Department of Computer Science Stanford University Stanford, CA United States; 4 Department of Neuroscience Stanford University Stanford, CA United States; 5 Department of Pediatrics Stanford University Stanford, CA United States; 6 Department of Psychology Stanford University Stanford, CA United States; 7 Department of Psychiatry and Behavioral Sciences Stanford University Stanford, CA United States; 8 Division of Systems Medicine Department of Biomedical Data Science Stanford University Palo Alto, CA United States

The authors of “Validity of Online Screening for Autism: Crowdsourcing Study Comparing Paid and Unpaid Diagnostic Tasks” (J Med Internet Res 2019;21(5):e13668) missed an important source of funding in the Acknowledgments section — Wu Tsai Neurosciences Institute Neuroscience: Translate Program. The revised Acknowledgments section now appears as follows:

We thank all the crowd workers and citizen scientists who participated in the studies. These studies were supported by awards to DW by the National Institutes of Health (1R21HD091500-01 and 1R01EB025025-01). Additionally, we acknowledge the support of grants to DW from The Hartwell Foundation, the David and Lucile Packard Foundation Special Projects Grant, Beckman Center for Molecular and Genetic Medicine, Coulter Endowment Translational Research Grant, Berry Fellowship, Spectrum Pilot Program, Stanford’s Precision Health and Integrated Diagnostics Center (PHIND), Wu Tsai Neurosciences Institute Neuroscience: Translate Program, and Stanford’s Institute of Human Centered Artificial Intelligence as well as philanthropic support from Mr. Peter Sullivan. HK would like to acknowledge support from the Thrasher Research Fund and Stanford NLM Clinical Data Science program (T-15LM007033-35).

The correction will appear in the online version of the paper on the JMIR website on June 27, 2019, together with the publication of this correction notice. Because this was made after submission to PubMed, PubMed Central, and other full-text repositories, the corrected article also has been resubmitted to those repositories.

