# Supplementary material for: Validity of Online Screening for Autism: Crowdsourcing Study Comparing Paid and Unpaid Diagnostic Tasks
Source: J Med Internet Res. 2019 May 23;21(5):e13668. doi: 10.2196/13668 (PMC6552453; doi:10.2196/13668)
Supplement: Multimedia Appendix 1 [file jmir_v21i5e13668_app1.pdf]

Full List of Questions

1. **Rate this child's display of echolalia (does the child immediately repeat the last statement made by the parent/caregiver, for example, repeating the parent's question? Do not include repetition that is appropriate to use in conversation or that is requested by the parent, i.e. "Say, 'dog.'").**

- o No demonstration of echolalia. Does not repeat others' speech.
- o Occasionally or rarely echoes others.
- o Mixed: some regular echoing of words and phrases, but also some language.
- o Mostly echoed speech

2. **Rate this child's expressive language/ conversation ability:**

<u>Clarification</u>: Expressive language is words used to communicate to others. Consider appropriate verbal skills for age when rating this question.

- o Excellent: Child is able to have a back and forth conversation, child responds to questions, and elaborates on their responses. Child may spontaneously address another person to engage in conversation.
- o Good: Child is able to respond to questions from examiner AND elaborates on their responses sometimes. Child engages individual(s) in conversation at times. Child elaborates and engages with individual(s) during conversation, but somewhat less than would be expected for their expressive language level and/or age.
- o Satisfactory: The child maintains very little back and forth conversation. Child may only follow their own train of thought, and not respond and stay on topic with others speaking to them. Child may address others spontaneously at times, but there is little engagement in back and forth conversation from the child.
- o Poor: The child rarely or never attempts to communicate with others in the video. Child may speak often in the video, but most of their language is not aimed at communicating with others (i.e. may be echoed speech, or the child may speak to themselves). Child may make some limited responses to the examiner (for example, saying "yes" or "no" with no elaboration).
- o N/A: The parent makes no attempts to initiate a conversation with the child.

3. **Rate this child's speech patterns.**

*<u>Clarification</u>: Abnormal speech includes: a*bnormal volume, pitch, intonation, rate, rhythm, stress, prosody or volume in speech. Consider appropriate language abilities for the child's age while rating.

- o Child has no abnormal qualities to speech and has appropriate volume, intonation, rhythm, rate, pitch, etc. when speaking.
- o Child has slightly unusual qualities to speech. For example, speaking too softly or too loudly, monotone or exaggerated intonation at times, speech that is too fast or too slow.
- o Child's speech is abnormal. The child's speech patterns make them hard to understand, due to odd volume, tone, rate/rhythm, intonation. Speech could be extremely monotone, pitch that seems unnatural (too high or too low), rhythm and pitch that seems robotic, or overly animated.
- o Stutter or stammer, and probable speech delay/disorder.
- o N/A: The child does not vocalize

4. Rate this child's subtle change in gaze, facial expression, and gesture while the child vocalizes. Include vocalizations used to maintain interaction or to respond to a person, as well as initiations

- o Child's vocalization is paired with appropriate facial expression, gesture, gaze.
- o Child's vocalization is paired with abnormal, limited frequency and/or range of gesture, gaze and facial expression. Child may only rely on only gesture, or only gaze, or only vocalization.
- o Child rarely or never links their vocalization with nonverbal communication, i.e. no or minimal use of gestures, facial expression, or socially directed gaze.
- o Child may avoid direct gaze, but could be attributed to shyness. Shows some use of nonverbal communication linked with vocalization.
- o N/A: The child does not demonstrate ANY body language linked with any kind of vocalization.

5. Rate the child's behaviors that involve any kind of aggressive act to self, even if not clearly harmful. This includes behaviors such as biting his/herself, hitting his/her head, or pulling his/her hair.
- Does not harm him/herself.
- Unclear. Some possible self-injury, and/or very rare self-injury.
- At least one clear example of self-injury.

6. Does the child entertain [himself/herself] or find things to do without others help?
- Yes, always entertains him/herself.
- Often does this.
- Sometimes does this.
- Rarely or never entertains him/herself.
- NA: Child has no opportunity to engage in any activity.

7. Does the child understand spoken language
- Yes, seems to always understand.
- Seems to often understand.
- Sometimes understands.
- Rarely or never demonstrates understanding.
- NA: not enough content in the video to answer.

8. Rate this child's use of appropriate eye contact. Appropriate eye contact is eye contact that appears natural (i.e. not staring at a person the entire time, but making and disengaging eye contact naturally and flexibly). If the child is shy initially, then slowly becomes more comfortable and begins making more socially appropriate eye contact, base ratings on their later behavior. If eye contact never improves, rate accordingly.
- Yes, always does this. Exhibits clear, flexible gaze that is linked with communication.
- Often makes socially-directed eye contact.
- Sometimes makes socially-directed eye contact.
- Rarely or never makes socially-directed eye contact.
- NA: not enough content in the video to answer.

9. Does the child look up and pay attention to the examiner or parent/caregiver when they talk to [him/her] without having to call the child's name?
- Yes, always pays attention without having to call name.
- Often does this, but is sometimes distracted.
- Sometimes does this, mixed with attention when name is called.
- Rarely or never does this – child only pays attention if name is called (or doesn't pay attention at all).
- NA: not enough content in the video to answer.

10. Does the child display any developmental challenges? (A developmentally appropriate 3-year-old child can consistently use multiword phrases and carry on a conversation with 2-3 sentences. Follows 2-step directions, e.g. "pick up your coat and bring it to me." Most speech is understandable. Sorts objects by shape and color. Imitates people or actions, and plays make-believe with toys or people. Climbs and runs well. A 2-year old uses 2-4 word sentences, follows simple instructions, begins sorting objects by color or shape, and copies adult's actions.)
- Met all milestones. No behavior indicating developmental delay.
- Uncertainty regarding behavior when compared with children the same age. Child may still be developmentally appropriate, but development is not clearly "normal" in the video to determine.
- Some developmental delays are demonstrated, with some minor or moderate departures from established milestones.
- Definite developmental delays, but not obvious as autism. Implies presence of another developmental delay.
- NA: not enough content in the video to answer AND/OR unsure

11. Based on what you've seen in this video, would the child comfort another person with words or actions if that person was not feeling well, hurt, or sad?

- Yes, would definitely do this.
- Probably would do this (50/50 chance).
- Unlikely.
- Almost certainly would not do this.
- NA: not enough information to tell (too short, etc).

12. Does the child seem to enjoy participating in social games and/or social interactions?

- Yes, clearly enjoys being engaged in social activities.
- Often does this, but limited.
- Sometimes demonstrates enjoyment, but does not spontaneously participate in interaction.
- Rarely or never demonstrates enjoyment in any social interaction.
- NA: not enough content in the video to answer (i.e., too few opportunities to demonstrate).

13. Does the child get upset, angry or irritated by particular sounds, tastes, smells, sights or textures?

- Yes, always gets upset or irritated by sensory stimulation.
- Often does this.
- Sometimes does this.
- Rarely or never does this.
- NA: not enough content in the video to answer--there is absolutely no sensory information surrounding the child

14. Does the child imitate others' actions, or would the child imitate others if given the chance?

- Yes, would definitely do this.
- Probably would do this (50/50 chance).
- Unlikely that the child would do this.
- Almost certainly would not do this.
- NA: Not enough information to tell (video too short, etc.)

15. Can you tell from the look on [his/her] face that the child is happy, surprised, sad, angry, afraid, guilty or embarrassed?

- Yes, always clearly expresses emotions in facial expressions.
- Often expresses emotions.
- Sometimes expressive.
- Rarely or never demonstrates variation in facial expression.
- N/A: The child's face is not visible.

16. Does the child stare at objects for long periods of time or focus on particular sounds, smells or textures, or like to sniff things?

- Yes, very drawn to sensory stimulation.
- Often does this.
- Sometimes does this.
- Rarely or never does this.
- NA: not enough content in the video to answer--there is absolutely no sensory information surrounding the child.

17. Does the child play pretend games when he/she plays with his/her toys?

- Yes, always does this.
- Often does this.
- Sometimes does this.
- Rarely or never does this.
- NA: Not enough content in the video to answer.

18. Does the child shake his/her head "no" or nod his/her head "yes" when asked a question or a request is made?

- Yes, always does this.
- Often does this.
- Sometimes does this.
- Rarely or never does this.
- NA: No questions or requests are made to the child.

19. Does the child like to share his/her excitement with others about things he/she likes or that make him/her happy? (for example: shows toy or drawing to parent)
- Yes, always does this.
- Often does this.
- Sometimes does this.
- Rarely or never does this.
- NA: not enough content in the video to answer.

20. Rate the child's responsive social smile.
- Excellent: Clearly smiles in response to another person's smile and/or playful verbal interaction.
- Good: Smiles in response to another person's smile/and or playful verbal interaction although stilted.
- Satisfactory: Smiles only after physical interactions or upon request.
- Poor: Does not smile at people but may smile at other things.
- N/A: This video does not provide sufficient data to accurately answer this question.

21. Does the child show or deliberately orient an object to call other's attention to things of interest
- (0) Yes. With accompanied eye contact.
- (1.5) Yes. Without accompanied eye contact.
- (3) No. The child has the opportunity to show or direct other's attention but does not do so.
- N/A: There is no opportunity to show or direct another's attention to an object.

22. Rate the child's attempt to draw another person's attention to objects or people that neither of them is touching. Do not include attempts that are made for the purpose of requesting
- (0) Excellent: Shifts gaze between object/person of interest to another person and back to the object/person (3- Point Gaze)
- (1) Satisfactory: Shifts gaze between object/person of interest to another person but not back to object/person (2- Point Gaze)
- (3) Poor: Does not show spontaneous joint attention.
- N/A: No objects in distance, and no opportunity for pointing.

23. Rate the child's play with objects (includes utensils, crayons, etc.).
<u>Clarification</u>*: Is the child playing appropriately or inappropriately with toys and/or objects?*
- Excellent: Clear spontaneous appropriate play.
- Good: Some spontaneous appropriate play.
- Satisfactory: Some appropriate play but does not engage spontaneously.
- Poor: Stereotyped or inappropriate play only.
- N/A: This video does not provide sufficient data to accurately answer this question.

24. Rate the child's creativity in his or her play and/or conversations
- Excellent: Clear demonstration of creativity.
- Good: Some demonstration of creativity
- Satisfactory: Some demonstration of creativity but not spontaneous.
- Poor: Does not demonstrate creativity.
- N/A: This video does not provide sufficient data to accurately answer this question.

25. Does the child use stereotyped words or phrases? Stereotyped words could include repeating lines from a movie, or repetitive language that is inappropriate in the social context. Odd uses of words or phrases include using the incorrect pronoun ("she/he" instead of "you"), made-up words, sentences that don't make sense in the context, or referring to oneself in the third person.
- No: Rarely or never uses stereotyped words or phrases.

- Yes: Some repetitive or odd use of words.
- Yes: Uses odd words or stereotyped language often, but not all language is stereotyped.
- Yes: Almost exclusively uses odd or stereotyped utterances.
- N/A: This video does not provide sufficient data to accurately answer this question.

26. Rate the child's spontaneous gestures
Clarification: Descriptive gestures (e.g., acting out rinsing a toothbrush or showing how a roller coaster curves through the air), Conventional gestures (e.g., clapping for a job well done), Instrumental gestures (e.g., shrugging, head nodding, head shaking). Exclude pointing and emphatic gestures (e.g., foot stomping). Descriptive gestures = acting out an action, doesn't have to be meshed with language. Conventional gestures = waving hello/goodbye, Informational gestures = how old are you, holds up 3 fingers/how big is that dog, moves arms to show how big Instrumental gestures = raising a hand, raising arms to get picked up Include: Spontaneous gestures, unprompted but can be in response to a question. Exclude: Pointing
- Excellent: Spontaneous use of at least one descriptive gesture. Conventional and instrumental gestures may be present as well.
- Good: Spontaneous use of more than one conventional and/or instrumental gesture but no descriptive gestures.
- Satisfactory: Spontaneous use of one conventional or instrumental gesture but no descriptive gestures.
- Poor: Does not spontaneously use gestures.
- N/A: No opportunity for gestures or child is limited by physical disability

27. Rate the child's ability to indicate pleasure with others
- (0) Excellent: Clearly expresses pleasure in interactions with others.
- (1) Satisfactory: Expresses pleasure in interactions with others although limited in amount or quality.
- (3) Poor: Does not express pleasure in interaction with others but may express pleasure in his/her own actions or does not show interest in others around them.
- N/A: No opportunity for social interaction.

28. Rate the quality of the child's initiations of social interactions. Can be verbal or nonverbal (i.e. raising arms to ask for hug).
- Excellent: Initiation of social interaction appropriate to context.
- Good: Slightly odd quality to initiations of social interactions.
- Satisfactory: Initiations are often inappropriate to the social context.
- Poor: Child does not initiate any social interactions.
- N/A: No opportunity to interact with another person (i.e. person filming child is too far away to communicate).

29. Does the child display hand and finger and other complex mannerisms, such as hand tensing, hand flapping, or holding up hands and looking intensely through the finger gaps?
- (0) No mannerisms are observed.
- (1) Yes, brief or unclear mannerisms.
- (3) Yes, definite mannerisms observed.
- N/A: This video does not provide sufficient data to accurately answer this question.

30. Does the child display inflexible stereotyped interests (focuses only on subject of interest to them, spinning wheel on toy, lining up toys) or repetitive behaviors (i.e. rocking, spinning, tapping)?
- No. Repetitive or stereotyped behaviors not observed.
- Yes. Behaviors observed briefly.
- Yes. Behaviors clearly observed but attention is also directed to other objects or activities.
- Yes. Behaviors observed the entire time. The child is rigid in his/her focus on the interest or behavior and may become upset if interrupted.
- N/A: This video does not provide sufficient data to accurately answer this question.

31. Do you think the child has autism?
- No, I am confident the child does not have autism
- No, but I am unsure

- Yes, but I am unsure
- Yes, I am confident the child has autism

Demographic Questions

What is your age (years)?
Where are you located? (City, State if applicable, and Country)
What is/are your occupation(s) (including "MTurk Worker")? Be as specific as possible without providing identifiable information. For example, instead of "teacher", you would write "9th grade history teacher".
What is your gender?
How many people with Autism do you know (in person)?
How many immediate family members with Autism do you have?
Are you a parent?
How many children with Autism do you have? (Write "N/A" if not a parent)
How many children with Autism do you know?
How many people with other behavioral conditions (such as ADHD, delayed speech, etc.) do you know?
Do you have autism?
